# The Emerging Role of miRNAs in HTLV-1 Infection and ATLL Pathogenesis

**DOI:** 10.3390/v7072805

**Published:** 2015-07-20

**Authors:** Ramona Moles, Christophe Nicot

**Affiliations:** Department of Pathology and Laboratory Medicine, Center for Viral Oncology, University of Kansas Medical Center, 3901 Rainbow Boulevard, Kansas City, KS 66160, USA; E-Mail: rmoles@kumc.edu

**Keywords:** human, HTLV-I infections, T-lymphotrophic virus 1, leukemia-lymphoma, adult T-cell, microRNAs, virus replication, cell line, cell transformation, gene expression regulation

## Abstract

Human T-cell leukemia virus (HTLV)-1 is a human retrovirus and the etiological agent of adult T-cell leukemia/lymphoma (ATLL), a fatal malignancy of CD4/CD25+ T lymphocytes. In recent years, cellular as well as virus-encoded microRNA (miRNA) have been shown to deregulate signaling pathways to favor virus life cycle. HTLV-1 does not encode miRNA, but several studies have demonstrated that cellular miRNA expression is affected in infected cells. Distinct mechanisms such as transcriptional, epigenetic or interference with miRNA processing machinery have been involved. This article reviews the current knowledge of the role of cellular microRNAs in virus infection, replication, immune escape and pathogenesis of HTLV-1.

## 1. Introduction

The transmission of the human T-cell leukemia virus (HTLV-1) retrovirus requires close contact with infected T cells, and occurs from mother to child, predominantly through breastfeeding as well as through sexual contact and blood transfusion [1,2]. The HTLV-1 infection is also associated with other diseases, such as: a chronic and progressive neurologic disorder named HTLV-1-associated myelopathy/tropical spastic paraparesis (HAM/TSP), polymyositis, infective dermatitis, HTLV-1-associated arthropathy, and HTLV-1-associated uveitis [2,3]. According to the Shimoyama classification, adult T-cell leukemia/lymphoma (ATLL) can be distinguished into four subtypes: smoldering, chronic and acute leukemic forms and ATLL lymphoma [4]. The overall survival of ATLL with different regimens of chemotherapy is poor, ranging between 5.5 and 13 months in patients presenting acute leukemia or lymphoma [5]. HTLV-1 mediates T lymphocyte transformation using a multistep process in which the virus promotes genomic instability, accumulation of genetic defects, and chronic proliferation of infected cells [6]. The genome of HTLV-1 encodes common retrovirus structural and enzymatic proteins, Gag, Pro, Pol, and Env, and additional accessory and regulatory proteins such as Tax, Rex, P30, p12, p13, and HTLV-1 basic leucine zipper factor protein (HBZ). Tax and HBZ regulatory proteins have been reported to play a central role in regulation of viral and cellular genes that lead to proliferation of infected cells [7]. Tax is a transcriptional trans-activator that promotes the expression of genes linked to the 5′ long terminal repeat promoter (LTR) element of the HTLV-1 genome [8]. Tax induces genomic instability [9] and promotes cell-cycle progression, survival and growth of HTLV-1-positive T cells [10]. HBZ is involved in the proliferation of infected cells *in vitro* and *in vivo* and plays an essential role in oncogenesis mediated by HTLV-1 in late stages of the disease when Tax is not expressed [11]. Consistently, HBZ was found to be expressed in ATLL cells through the whole period of ATLL development, suggesting that it might be involved in maintenance of HTLV-1-transformed cells [12]. Rex is a post-transcriptional regulator of viral expression, which activates viral replication in the early phase of HTLV-1 infection by promoting the nuclear export of HTLV-1 mRNA [13]. Several studies have shown altered expression of microRNAs (miRNAs) in HTLV-1/ATLL cell lines and primary peripheral blood mononuclear cells (PBMCs) from ATLL patients, suggesting that miRNA deregulation is involved in HTLV-1 infection and adult T-cell leukemia/lymphoma pathogenesis. MicroRNAs play an essential role in a wide range of biological processes, including development, differentiation, cell cycle, apoptosis and oncogenesis [14,15,16].

## 2. MiRNA Biogenesis

MicroRNAs (miRNAs) are small, non-coding RNA molecules that transcriptionally regulate gene expression. The first miRNA identified in animals is *Lin-4*, discovered in 1993 by Ambros and colleagues. *Lin-4* was identified as heterochronic genes in *Caenorhabditis elegans* involved in cell fate [17,18]. Subsequent studies have shown the involvement of miRNAs in different biological processes, including tumorigenesis by targeting oncogenes or tumor suppressor genes [16]. MiRNA sequences are localized in different genomic contexts. Some miRNAs are encoded by exon; however, the majority are encoded by the intronic region of non-coding and coding transcripts [19]. MiRNAs are transcribed by the RNA polymerase II or III into the nucleus as primary miRNAs (pri-miRNAs). Pri-miRNAs are normally over 1 kilobase and contain a local steam-loop structure in which mature miRNA sequences are included. The nuclear RNase III Drosha recognized and processed pri-miRNAs into a hairpin-shaped RNA of nearly 65 nucleotides in length, named precursor miRNAs (pre-miRNAs). After transport to the cytoplasm by the RanGTP-dependent dsRNA-binding protein Exportin 5, pre-miRNAs are processed by the cytoplasmic RNase III Dicer, liberating a mature 20–24 nucleotide long duplex. Argonaute family proteins, AGO, and Trans-Activation Responsive RNA-Binding Protein (TARBP2), together with the duplex form a complex named RNA-Induced Silencing Complex (RISC) [19,20]. One strand of the duplex, called guide strand, is incorporated into the RISC complex while the other strand, named passenger strand, is targeted for degradation [21]. Apart from the canonical miRNA biogenesis described above, different alternative mechanisms, which bypass Drosha processing, were described [22]. MiRNAs can be generated through non-canonical pathways, wherein the precursor miRNAs are cleavaged by Dicer. Mirtrons represent an example of miRNA processed by a non-canonical pathway. They are generated from intron lariats serving as pri-miRNAs, which is processed by Spliceosome that function as Drosha, to release pre-miRNAs [22,23]. MiRNAs bind complementary sequences usually localized at 3′UTR of messenger RNA and guide RISC to target mRNA. MiRNAs used different mechanisms to regulate post-transcriptional gene expression: inhibition of translation and/or messenger RNA degradation. The repression of many miRNA targets is frequently associated with their destabilization. Degradation of target mRNA is characterized by gradual shortening of the mRNA poly-Adenine tail, which is catalyzed by the exosome or exonuclease XRN1. MiRNAs might also induce gene silencing by interfering with protein translation [24]. Several pieces of evidence show that miRNA silencing is observed with either no change in the mRNA level or with a significantly smaller decrease of mRNA compared to the protein level [25,26]. Deregulated MiRNAs in HTLV-1 context will be discussing in the next section of the review.

## 3. MiRNA Profile in HTLV-1-Transformed Cell Lines and ATLL Patients

Four studies have characterized miRNA expression profiles in HTLV-1/ATLL cell lines and ATLL patients. Pichler [27] and colleagues chose the phenotype of regulatory T cells (Treg) as a starting point to study miRNA expression in HTLV-1-transformed cells. The authors have selected and analyzed the expression of a set of miRNAs characteristic of murine Treg and downregulated in different tumors. The analysis identified five deregulated miRNAs: miR-21, miR-24, miR-146a, and miR-155 were found upregulated, whereas miR-223 was downregulated. Bellon [28] and colleagues analyzed miRNA profiles from ATLL patients compared to HTLV-1-negative donors by using microarray. The results were confirmed by Real Time (RT)-PCR of mature miRNAs in uncultured ATLL cells and HTLV-1-transformed cell lines. Microarray analysis and RT-PCR demonstrated downregulation of miR-181a, miR-132 and miR-125a and upregulation of miR-155 and miR-142-3p. This study identifies two miRNAs differently expressed *in vitro* and *in vivo.* MiR-150 and miR-223 were both upregulated in uncultured ATLL cells and downregulated in HTLV-1-transformed cell lines. Yeung [29] and colleagues examined miRNA profiles in several ATLL-derived cell lines and primary peripheral blood mononuclear cells (PBMCs) from acute ATLL patients using miRNA microarray. Several HTLV-1/ATLL cell lines and four ATLL patients were studied. Thirteen miRNAs were found to be upregulated and thirty downregulated among the different cell lines. In parallel, 22 upregulated and 22 downregulated miRNAs were identified in acute ATLL patients. Among those, miR-9, miR-17-3p, miR-20b, miR-93, miR-130b and miR-18a were found to be induced; in contrast, miR-1, miR-144, miR-126, miR-130a, miR-199a, miR-338, miR-432, miR-335 and miR-337 were found to be downregulated. Yamagishi and colleagues [30] studied the miRNA expression signature in primary ATL cells by using microarray analysis compared to CD4+ T cells from healthy donors. The results show that 59 of the miRNAs tested were found with a decrease in ATL primary cells. Among them, miR-31 was the one most profoundly repressed.

## 4. HTLV-1 Interferes with Cellular miRNA Machinery

**Figure 1:**
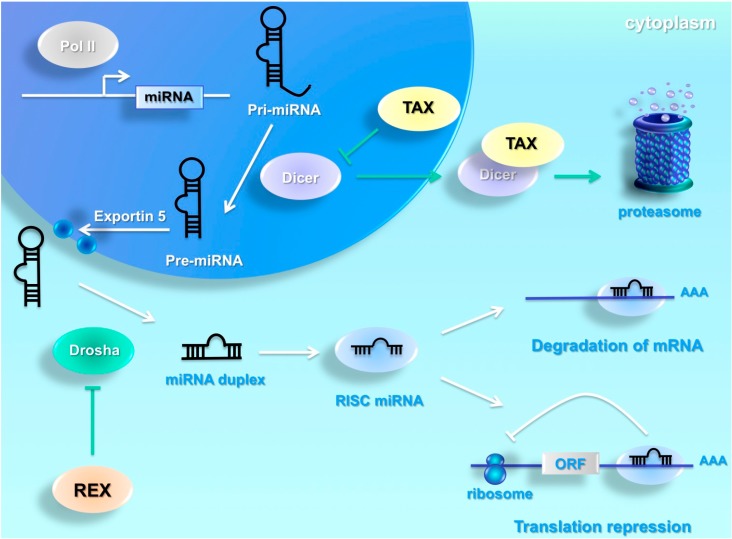
Human T-cell leukemia virus HTLV-1 interferes with cellular miRNA machinery. MiRNAs are transcribed by the RNA polymerase II or III into the nucleus as primary miRNAs (pri-miRNAs) from coding or non-coding part of genes. The nuclear RNase III Drosha recognized and processed pri-miRNAs into a hairpin-shaped RNA, named precursor miRNAs. Pre-miRNAs are transported to the cytoplasm by Exportin 5, and processed by the cytoplasmic RNase III Dicer in the mature miRNA duplex. The duplex forms a complex named RNA-Induced Silencing Complex (RISC). MiRNAs bind complementary sequences usually localized at 3′UTR of messenger RNA and this binding results in the inhibition of translation and/or messenger RNA degradation. HTLV-1 deregulates the cellular miRNA pathway by suppressing the function of Drosha and Dicer. Tax directly interacts with Drosha and the binding leads to Drosha degradation mediated by proteasome complex. The regulatory protein, Rex, is reported to directly interact with Dicer. Rex suppresses the ribonuclease-directed processing activity of Dicer, protecting against the cleavage Rex-mRNA.

The dysregulation of miRNA pathways has been reported across several viruses, including HIV, Ebola, Epstein–Barr, Influenza, HBV, HCV, Adenovirus, and HTLV-1 [31,32,33,34,35,36,37,38]. Drosha was reported to be downregulated in HTLV-1-infected cell lines, HTLV-1-transfected cells, and infected primary cells [38]. Van Duyne [38] and colleagues proposed that HTLV-1 deregulates the cellular RNAi pathway, including miRNAs, by suppressing the function and degrading Drosha (Figure 1).

The authors have demonstrated a direct interaction between the Tax oncoprotein and Drosha, which is responsible for its downregulation. The *N*-terminal region of Tax presents two putative motifs, the Zinc finger motif and leucine-zipper-like region, which interact with Drosha. The Tax *N*-terminal region is reported to interact with the proteasome complex. Van Duyne and colleagues demonstrated that the binding between Tax and Drosha leads to its degradation mediated by proteasome complex. In addition, Drosha increases HTLV-1 replication and is not efficient in processing miRNAs when Tax is expressed, suggesting that the dysregulation of miRNA machinery might be involved in the rate of HTLV-1 infection [38]. The HTLV-1 regulatory protein, Rex, is reported to directly interact with Dicer. Abe [39] and colleagues have demonstrated that Rex suppresses the ribonuclease-directed processing activity of Dicer, protecting against the cleavage Rex-mRNA (Figure 1). Inhibition of Dicer activation might represent an additional mechanism used by HTLV-1 to deregulate cellular miRNA expression.

## 5. MiRNAs Target the HTLV-1 Genome

**Figure 2:**
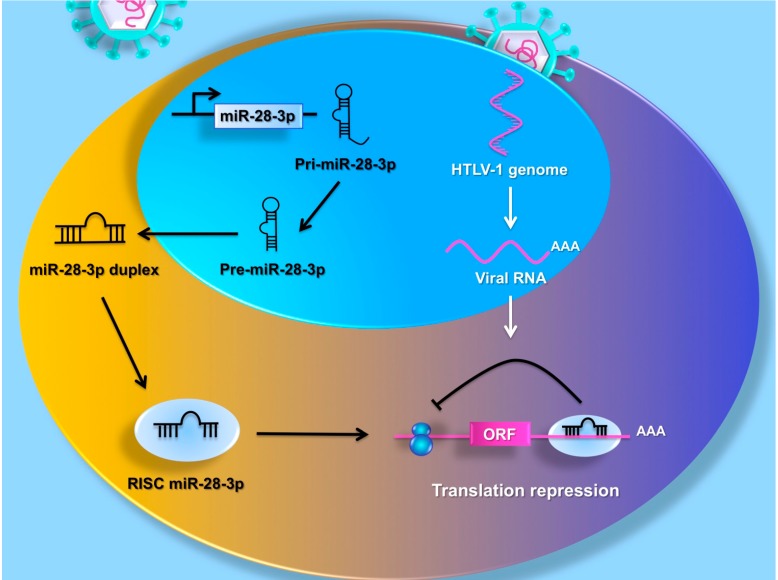
MiR-28-3p targets the HTLV-1 genome. The figure illustrates a natural feedback loop that regulated cellular miRNA expression in response to virus infection. MiR-28-3p suppresses HTLV-1 expression by targeting a sequence localized within the viral gag/pol HTLV-1 sequence. MiR-28-3p expression leads to abortive infection by inhibiting HTLV-1 reverse transcription and preventing the formation of the pre-integration complex.

The cellular environment has an essential role in virus infection and replication. Many cellular genes prevent replication and virus dissemination by acting as innate immunity factors. However, viruses have evolved strategies to avoid activation of an antiviral state: virus-derived miRNAs can enhance viral gene expression, replication, and infectivity [40] or suppress the IFN response [41]. The genome of the Epstein–Barr virus (EBV), Kaposi sarcoma-associated herpesvirus (KSHV), human cytomegalovirus (hCMV) and bovine leukemia virus (BLV) encodes for virus-derived miRNAs [42,43,44,45]. BLV shares many characteristics in disease pathogenesis with HTLV-1 and is associated with the development of B-cell tumors. Kincaid and colleagues [45] show that BLV is capable of producing miRNAs *in vitro*. A subsequent study demonstrated that BLV encodes a conserved cluster of miRNAs located in a specific BLV proviral region, which is essential for *in vivo* infectivity [46]. BLV has different common features in genomic organization with HTLV-1, however HTLV-1-encoded miRNAs have not been reported. Cellular miRNAs can promote virus replication or negatively regulate virus expression and infectivity [47]. MiR-28, miR-125b, together with miR-150, miR-223 and miR-382 target 3′ ends of HIV-1 messenger, promoting viral latency [47]. Bai and colleagues identified a binding site in the HTLV-1 genome for miR-28-3p and demonstrated a mechanism used by a cellular miRNA to prevent HTLV-1 gene expression and viral transmission (Figure 2) [48].

MiR-28-3p was found to target a sequence localized within the viral gag/pol HTLV-1 mRNA and reduced viral replication and gene expression. MiR-28-3p-expressing cells are characterized by reduced levels of HTLV-1 gag p19 and p24 products and they are resistant to infection. MiR-28-3p expression leads to abortive infection by inhibiting HTLV-1 reverse transcription and preventing the formation of the pre-integration complex. MiR-28-3p suppresses HTLV-1 expression and infection; this is consistent with the high levels of miR-28-3p reported in resting T cells and their inability to be infected by HTLV-1 without prior activation. Bai and colleagues [48] demonstrated a natural feedback loop that regulated miR-28-3p expression in response to virus infection. It is well established that *de novo* infection in T cells activates the interferon anti-viral response. MiR-28-3p expression was found to be induced after IFN-α or - γ stimulation, suggesting that miR-28-3p might contribute to restricting virus expansion to neighboring cells by reducing local inflammation and the initial establishment of latent infection. The miR-28-3p site is highly conserved in HTLV-1 subtypes B and C, at nearly 90%. However, the subtype 1A, Japanese ATK-1, presents a natural polymorphism (T to C substitution) within the miR-28-3p target site. The mutation is silent and more resistant to miR-28-3p inhibition of viral replication. Bai and colleagues [48] proposed a model where the modulation of miR-28-3p expression affected HTLV-1 virus spreading. Virus particles can transiently activate resting T cells by reducing miR-28-3p expression and favoring infection. Because IFN response is a potent inducer of miR-28-3p expression, the initial antiviral response might backfire, helping to protect newly infected cells from being eliminated by the immune system.

## 6. MiRNAs Promote Cell Proliferation

### 6.1. MiR-146a

**Figure 3:**
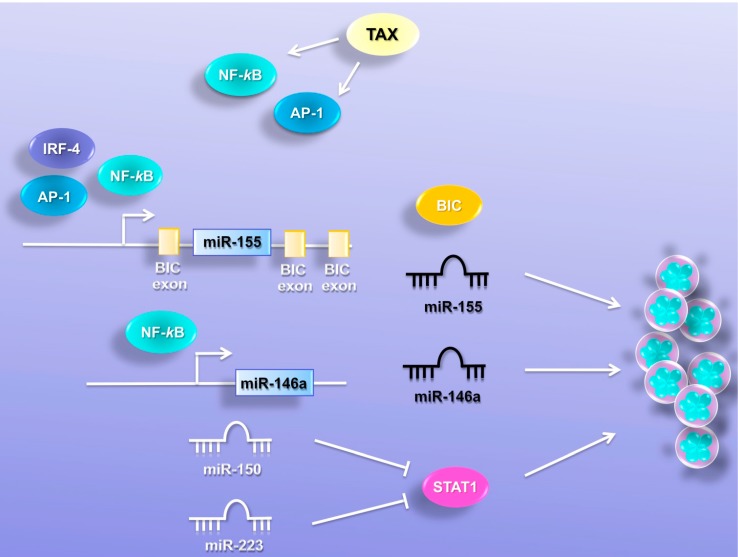
MiRNAs promote cell proliferation. MiR-155 and miR-146a were found elevated in HTLV-1-infected cells *in vitro.* Tax induces the transcription factors NF-κB and AP-1, which promote miR-155 expression by binding the miRNA promoter. This binding resulted in an increased expression of the B-cell integration cluster (BIC) gene whose transcript is processed into miR-155. The interferon regulatory factor-4, IRF4, which is induced in HTLV-1-infected cells, promotes BIC/miR-155 expression. NF-κB also mediates miR-146a transactivation; both miRNAs enhance cellular growth in HTLV-1-infected cells. MiR-150 and miR-223 are differentially regulated in ATLL samples and in HTLV-1-transformed cells. MiR-150 and miR-223 were found upregulated in acute ATLL patients and downregulated in HTLV-1-transformed cell lines. MiR-150 and miR-223 target the STAT1 3′UTR. Inhibition of STAT1 expression, through miR-150, miR-223 reduced proliferation of HTLV-1-transformed and ATLL-derived cell lines. MiR-150 and miR-223, by decreasing STAT1 expression and dampening STAT1-dependent signaling in human T cells, regulated proliferation in an HTLV-1 context.

MiR-146a has a central role in the regulation of immune response and its expression is induced by NF-κB signaling. MiR-146a is deregulated in different cancers. A high level of expression was reported in papillary thyroid carcinoma, anaplastic thyroid cancer, breast cancer, glioblastoma and cervical cancer [49,50,51,52,53]. In contrast, low-expressing levels were described in pancreatic carcinoma, gastric cancer, prostate cancer, acute myeloid leukemia (AML), myeloblastic syndrome and chronic myeloid leukemia (CML) [54,55,56,57,58,59]. MiR-146a was found to be upregulated in HTLV-1-transformed cell lines [27]. Ectopic expression of Tax in HTLV-1-negative T cells, Jurkat, induced miR-146a expression. Promotor analysis showed a 15-fold activation of miR-146a by Tax [60], suggesting that HTLV-1 infection might be involved in the regulation of miR-146a expression. Pichler [27] and colleagues used a mutated form of Tax and dominant active NF-κB inhibitor to show that miR-146a transactivation is mediated by NF-κB (Figure 3).

Tomita *et al.* [60] also reported an NF-κB binding site on the miR-146a gene. In addition, it has been described as having a suppressive effect of miR-146a on NF-κB signaling [51]. This might represent a negative feedback loop, which seems to be ineffective in HTLV-1-infected cells. MiR-146a has also been shown to induce proliferation in several human cancers, including cervical cancer [53], breast cancer cells [61], gastric cancer cells [62] and mesenchymal stem cells (MSCs) [63]. Consistent with this report, treatment with an anti-miR-146a inhibitor suppressed the proliferation of HTLV-1-transformed cell lines but not uninfected T-cell lines. In addition, overexpression of miR-146a increased the growth of HTLV-1-transformed cell lines [27]. Because overexpression of miR-146a has also been described in an EBV context, Tomita [60] and colleagues suggested that miR-146a up-regulation might represent a common mechanism in the pathogenesis of persistent viruses. Wang *et al.* [64] identified 622 putative target genes of miR-146a that are predicted by using different prediction programs. Gene ontology analysis shows that these genes are involved in the inhibition of cell growth and promotion of apoptosis, and this partially explains the role of miR-146a in the proliferation of HTLV-1-transformed cells.

### 6.2. MiR-155

MiR-155 has been implicated in normal hematopoiesis [65], immune response [66], and in the carcinogenesis of different human tumors [67,68]. Mouse studies have reported that transgenic overexpression of miR-155 results in the increased frequency of tumor formation [69]. Overexpression of miR-155 was found in breast cancer [70], pancreatic cancer [71], lung cancer [72], B-cell lymphoma [67], MALT lymphoma [73] and acute myeloid leukemia (AML) [74]. MiR-155 was found elevated in HTLV-1-infected cells *in vitro* and *in vivo* [28,75], suggesting that this miRNA might play an important role in the biology and pathogenesis of HTLV-1. Babar [76] and colleagues used an inducible knock-in mouse model to show that miR-155 induction in the lymphoid tissue led to disseminated lymphoma. In contrast, reduction of miR-155 resulted in the decrease of tumor size. In humans, Calin [77] and colleagues identified a miRNA signature associated with progression and prognosis in chronic lymphocytic leukemia (CLL) and showed an association between miR-155 upregulation and poor prognosis. Several lymphoma-associated viruses, including the Epstein-Barr virus, Kaposi sarcoma-associated herpesvirus and Marek’s disease virus, are characterized by overexpression of miR-155 [73,78], suggesting that HTLV-1 infection might be responsible for the induction of miR-155 in infected T cells. MiR-155 upregulation has been reported in HTLV-1 cell lines and ATLL patients [28,75]. Tomita [75] and colleagues demonstrated that transcription factors NF-κB and AP-1 induced miR-155 expression by binding the miRNA promoter in an HTLV-1 context (Figure 3). This binding resulted in an increased expression of the B-cell integration cluster (*BIC*) gene whose transcript is processed into miR-155 (Figure 3). Tomita and colleagues demonstrated that miR-155 overexpression enhanced the growth in HTLV-1-transformed cells. Consistently, treatment with anti-miR-155 reduced the proliferation of these cells and had no effect on HTLV-1-negative T cells. Wang [79] and colleagues demonstrated that interferon regulatory factor-4, IRF4, which is reported to be oncogenic [80], induces BIC/miR-155 expression in HTLV-1-transformed cells (Figure 3). In normal lymphocytes, IRF4 is involved in cellular proliferation and differentiation [80]. In mature human CD4+ T cells, IRF4 is essential for cytokine production and survival [81,82]. Several studies show that IRF4 is overexpressed in HTLV-1-transformed and primary ATLL/L cells and associated with poor prognosis [81,82,83], suggesting that IRF4 might be involved in HTLV-1 pathogenesis. Wang and colleagues show that depletion of IRF4 drastically reduced cell proliferation of HTLV-1-transformed cell lines, suggesting that the IRF4/miR-155 pathway might play a central role in the malignant proliferation of HTLV-1-infected cells [80]. In addition, miR-155 is reported to target Tumor Protein 53-Induced Nuclear Protein 1 (TP53INP1) in liver cancer stem cells [84], which promotes cell cycle arrest and apoptosis, suggesting a possible mechanism that could enhance cellular proliferation in an HTLV-1 context.

### 6.3. MiR-150 and MiR-223

MiR-150 and miR-223 were reported to be differentially regulated in HTLV-1-transformed cells and in ATLL samples. MiR-150 and miR-223 were found upregulated in acute ATLL patients and downregulated in HTLV-1-transformed cell lines, suggesting that different selective pressure *in vitro* and *in vivo* might regulate the expression of those miRNAs. MiR-150 can have either oncogenic or tumor suppressor activity in different human tumors. It is overexpressed in chronic lymphocytic leukemia (CLL) [85,86] and downregulated in chronic myeloid leukemia (CML) [87,88], acute lymphoblastic leukemia (ALL) [89] and mantle cell lymphoma (MCL) [90]. Additional studies show that miR-150 promotes the proliferation and migration in lung cancer by targeting SRC kinase signaling inhibitor 1 (SRCIN1) and SRC activity [91]. In contrast, miR-150 expression was reported to inhibit cell migration and invasion in breast cancer [92,93]. C-MYB, NOTCH3, CBL, EGR2, AKT2 and DKC1 are established targets of miR-150 [94,95,96,97,98]. MiR-223 was reported to be differentially regulated in human cancers; it is downregulated in hepatocellular carcinoma, B-cell chronic lymphocytic leukemia (B-CLL), acute myeloid leukemia (AML), gastric MALT lymphoma and recurrent ovarian cancer [99,100,101,102,103]. In contrast, miR-223 is upregulated in T-cell acute lymphocytic leukemia (T-ALL), EBV-positive diffuse large B-cell lymphoma, and metastatic gastric cancer [104,105,106,107,108]. FBXW7/Cdc4, RhoB, STMN1, E2F1, STAT3, C/EBPβ, FOXO1 and NFI-A are validated targets of miR-223 [106,107,108,109,110,111]. It has previously been shown that E2F1 represses the miR-223 promoter [110,111,112]. Interestingly, viral HBZ mRNA increases the expression and transcriptional activity of E2F1. HBZ expression is consistently increased in ATLL cells *in vivo* [11]. These observations can partially explain the differential regulation of miR-223 *in vitro* and *in vivo*. MiR-150 and miR-223 target the STAT1 3′UTR in an HTLV-1 context (Figure 3). STAT1 plays an essential role in immune modulatory functions, anti-viral responses, apoptosis and anti-proliferative responses [113]. In addition, several studies have shown that STAT1 can also act as a potent tumor promoter for leukemia development [114] and that many T-ALL leukemic cells are dependent upon the TYK2-STAT1-BCL2 pathway for continued survival [115]. Inverse correlation between STAT1 expression and miR-150 and miR-223 was identified in HTLV-1-transformed and IL-2-independent ATLL-derived cells [116]. IL-2-dependent ATLL cells display a high level of miR-150 expression, but low miR-223, suggesting that miR-150 might be regulated through the IL-2 signaling pathway. Absence of IL-2 signaling results in miR-150 downregulation in IL-2-dependent ATLL cells. In contrast, IL-2 stimulation in IL-2-independent ATLL-derived cells leads to miR-150 induction. This evidence suggests that miR-150 is regulated by the IL-2 signaling pathway. It was reported that ATLL tumor cells *in vivo* produce IL-2 or IL-15 and express IL-2 receptor alpha chain, CD25. These observations partially explain the higher levels of miR-150 in ATLL patients compared with HTLV-1 cell lines. Despite the miR-150 and miR-223 overexpression in freshly isolated ATLL samples, STAT1 was found to be induced in a majority of ATLL samples, suggesting that miR-150 and miR-223 cannot efficiently suppress STAT1 expression in ATLL patient cells. STAT1 has been reported to have tumor promoting activities. Inhibition of STAT1 expression, through miR-150, miR-223 or directly by shRNA targeting, reduced proliferation of HTLV-1-transformed and ATLL-derived cell lines. MiR-150 and miR-223, by decreasing STAT1 expression and dampening STAT1-dependent signaling in human T cells, regulated proliferation in an HTLV-1 context.

## 7. MiRNAs Induce Resistance to Apoptosis

### 7.1. MiR-31

**Figure 4:**
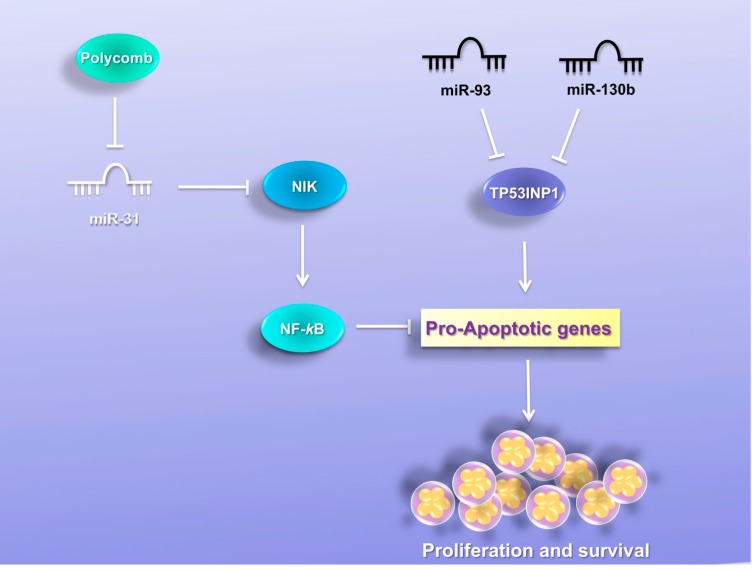
MiRNAs induce resistance to apoptosis. MiR-31 is one of the most profoundly repressed miRNAs in primary ATLL cells. The Polycomb protein complex is overexpressed in ATLL cells and suppresses miR-31 expression. MiR-31 negatively regulates NF-κB-inducing kinase (NIK) and leads to apoptosis resistance. MiR-130b and miR-93 are upregulated in HTLV-1 cell lines and ATLL patients and both target Tumor protein p53-inducible nuclear protein (TP53INP1). TP53INP1 is a tumor suppressor gene that has anti-proliferative and pro-apoptotic activities via both p53-dependent and p53-independent means. TP53INP1 has in its 3′ UTR two binding sites for miR-93 and two sites for miR-130b.

Yamagishi [30] and colleagues identified miR-31 as one of the most profoundly repressed miRNAs in primary ATLL cells. MiR-31 is reported as a tumor suppressor and correlates inversely with metastasis in breast cancer [117]. MiR-31 *in vivo* targets several genes, such as Fzd3, ITGA5, MMP16, RDX, RhoA, WAVE3 and integrin α5 subunit, that contribute to cell migration and metastatic invasion [117,118]. The Polycomb protein complex has been reported to be a strong suppressor of miR-31 in breast cancer and adult T-cell leukemia [30,117]. Polycomb group proteins are overexpressed in ATLL cells [119] and have an important role in cellular development and regeneration by controlling histone methylation, especially at histone H3 Lys27 (H3K27), which induces chromatin compaction. The Polycomb family is associated with cancer phenotypes and malignancy in breast cancer, prostate cancer, bladder tumors, and other neoplasms [120,121]. MiR-31 negatively regulates NF-κB-inducing kinase (NIK) expression and activity in adult T-cell leukemia and other cancers [30]. NIK has an important role in tumor progression and the aggressive phenotypes of various cancers. It is well established that the NIK level directly regulates NF-κB activity in various cell types [122]. Constitutive activation of the nuclear factor NF-κB is observed in the ATLL cell lines and primary isolated tumor cells from ATLL patients [123]. NF-κB activation contributes to cell propagation and anti-apoptotic responses in ATLL [124]. An inverse correlation has been reported between the expression level of miR-31 and NIK in ATLL patients. Rescue of miR-31 represses NF-κB expression and leads to increased proliferation and apoptosis resistance. The inhibition of NF-kB promotes tumor cell death in HTLV-1-transformed cells and primary ATLL cells. The model proposed by Yamagishi and colleagues show that the Polycomb group regulates miR-31 expression and leads to NF-κB activation via NIK-miR-31 regulation and apoptosis resistance in HTLV-1 context (Figure 4). The downregulation of miR-31 might play an important role in ATLL pathogenesis.

### 7.2. MiR-130b and MiR-93

Microarray analyses demonstrated that miR-130b and miR-93 were consistently upregulated in HTLV-1 cell lines and ATLL patients and both target Tumor protein p53-inducible nuclear protein, TP53INP1 [29]. MiR-130b was found to be deregulated in several human cancers. Overexpression of miR-130b has been reported in colorectal cancer, gastric cancer, bladder cancer, cutaneous malignant melanoma, and head and neck squamous cell carcinoma [125,126,127,128]. In contrast, miR-130b is downregulated in papillary thyroid carcinoma, ovarian cancer and endometrial cancer [129,130,131,132]. Identified targets of miR-130b are STAT3, PTEN and TGF-b1 [133,134,135]. MiR-93 belongs to the miR-106b-25 cluster, which also includes miR-106b and miR-25 [136]. The miR-106b-25 cluster is overexpressed in neuroblastoma, multiple myeloma, and lung, prostate and gastric tumors [136,137,138]. Reported targets of miR-93 are PTEN, VEGF, ITGB8, DAB2 and LATS2 [139,140,141,142,143]. TP53INP1 is a tumor suppressor gene that has anti-proliferative and pro-apoptotic activities via both p53-dependent [144] and p53-independent means [145]. TP53INP1 has in its 3′ UTR two binding sites for miR-93 and two sites for miR-130b. Yeung [29] and colleagues have shown that transfection of antagomirs against miR-93 and miR-130b into an HTLV-1-transformed cell line increased the expression of TP53INP1 and decreased cellular viability by promoting apoptosis (Figure 4). These results show that TP53INP1 has anti-proliferative properties and can be regulated by miR-130b and miR-93. Transfection of miR-93 or miR-130b in HTLV-1-negative T-cell lines reduced TP53INP1 expression and increased cellular proliferation. It has been reported that loss of TP53INP1 correlates with the development of cancers [146,147] and its induction promotes G1 cell cycle arrest and apoptosis [144,145,148]. This evidence suggests that up-regulation of miR-130b and miR-93 reduces TP53INP1 levels in ATLL cells and promotes cellular proliferation. TP53INP1 is also able to reduce cell migration in pancreatic cancer cells [149] and this might be significant because it is well established that HTLV-1 infection promotes T-lymphocyte migration [150].

## 8. MiRNAs Promote Chromatin Remodeling

**Figure 5:**
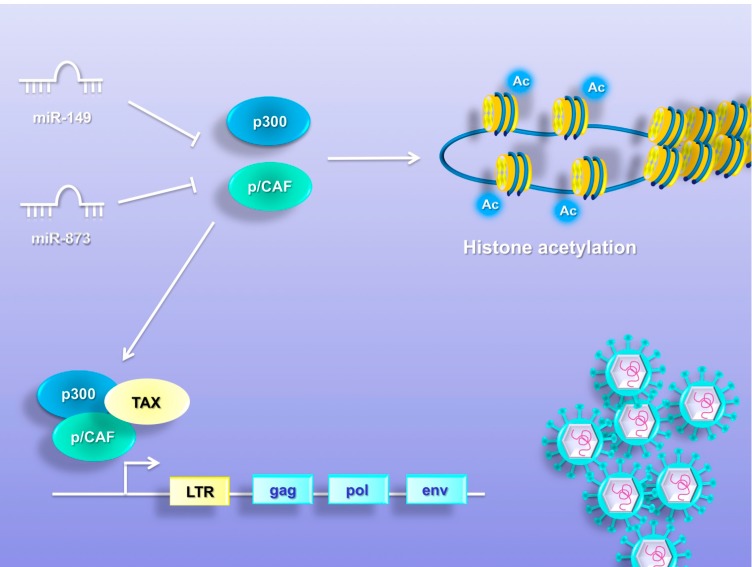
MiR-149 and miR-873 promote chromatin remodeling. The Tax protein promotes HTLV-1 gene expression by its interaction with the long terminal repeat (LTR) or U3 region of the viral promoter. To activate the transcription, Tax recruits the p300/CREB-binding protein (p300/CBP) and p300/CBP-associated factor (P/CAF), which bind two different regions of Tax, resulting in histone acetylation and chromatin remodeling. MiR-149 and miR-873 are downregulated in HTLV-1-transformed cell lines and target the chromatin remodeling factors p300 and p/CAF.

The Tax protein promotes HTLV-1 gene expression by its interaction with the long terminal repeat (LTR) or U3 region of the viral promoter [151,152]. To activate the transcription, Tax recruits the p300/CREB-binding protein (p300/CBP) and p300/CBP-associated factor (P/CAF), which bind two different regions of Tax, resulting in histone acetylation and chromatin remodeling (Figure 5) [153,154,155,156,157,158]. Rahman [157] and colleagues identified the chromatin remodeling factors, p300 and p/CAF, as a target of miR-149 and miR-873. MiR-149 has been reported to have a role as an oncogene and tumor suppressor in different human cancers [158,159]. Downregulation of miR-149 has been described in prostatic cancer, astrocytomas and renal carcinoma [160,161,162]. In contrast, miR-873 was found to be suppressed in colorectal cancer, glioblastoma and breast cancer [163,164,165]. Recent evidence has established the role of miR-873 in cell proliferation, tumor growth and tamoxifen resistance in breast cancer [165]. MiR-149 and miR-873 were found to be profoundly downregulated in HTLV-1-transformed cell lines, MT-2, compared to an uninfected control, Jurkat [157]. To verify that miR-149 and miR-873 could target p/CAF and p300, the authors over-expressed these miRNAs in HTLV-1-transformed cells and observed a significant reduction in the expression of chromatin-remodeling enzymes. In addition, the cell culture supernatant was analyzed for viral protein p19 before and after transfection. The results show a decrease in the levels of viral progeny production in cells transfected with miR-149 and miR-873, suggesting that these miRNAs, by targeting chromatin remodeling factors p/CAF and p300, might play a role in HTLV-1 infection and pathogenesis (Figure 5).

## 9. MiRNAs Induce Genetic Instability

**Figure 6:**
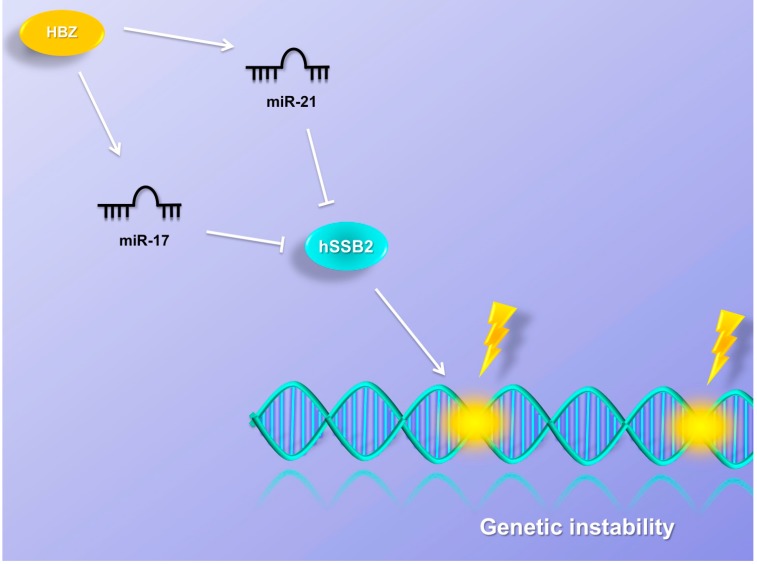
MiRNAs induce genetic instability. MiR-17 and miR-21 are upregulated in an HTLV-1 context. HBZ inactivates OBFC2A via miR-17 and miR-21, promoting genetic instability and cell proliferation. OBFC2A encodes for hSSB2, which is involved in the ATM signaling pathway, the activation of the cell cycle checkpoint and promotes DNA repair.

MiRNA expression analysis in CD4+ lymphocytes, derived from HAM/TSP patients, has identified a high expression level of miR-17 and miR-21 [166]. Spry 1, Spry 2, PTEN, TPM1 and Pdcd4 have been reported to be miR-21 targets, suggesting its central role in cell proliferation, apoptosis, and invasion [167,168,169,170,171]. MiR-17, instead, is the main effector of the miR-17-92 cluster component, which has been identified as a member of the miRNA signature in solid tumors [172]. MiR-17 regulates E2F1 and c-Myc, p21, PTEN and BIM expression [173,174,175,176], suggesting its potential functions in cell migration, invasion and proliferation. Vernin [166] and colleagues identified OBFC2A as a potential target of miR-17 and miR-21 in an HTLV-1 context. OBFC2A encodes for hSSB2, which is involved in the ATM signaling pathway, the activation of the cell cycle checkpoint and promotes DNA repair. The down-regulation of OBFC2A and a positive correlation between miR-17, miR-21 and HBZ expression has been reported in HTLV-1-infected cells [166]. Vernin and colleagues suggested that HBZ inactivates OBFC2A via miR-17 and miR-21, promoting genetic instability and cell proliferation (Figure 6).

The authors have shown that ectopic expression of HBZ does not decrease cellular growth in DNA-damaged cells. HBZ-expressing cells continued to proliferate when treated with a DNA-damaging agent, neocarzinostatin. This phenotype can be reversed by ectopic expression of OBFC2A, which leads to a decrease of proliferation rates and restores the DNA damage response. This evidence suggested a potential role of miR-17 and miR-21 in genetic instability and cell proliferation in HTLV-1-infected cells.

## 10. Conclusions and Prospective

The role of miRNAs in HTLV-1 infection and ATLL pathogenesis is beginning to emerge. Available evidence shows a complex interplay between cellular miRNA machinery and virus infection. HTLV-1 inhibits proteins involved in biogenesis and maturation of cellular miRNAs, resulting in a perturbation of the expression profile of host miRNAs. In this review, we focused on miRNAs, which are involved in virus production, establishment of latency, tumor cell transformation and proliferation. A potential role of MiRNA modulation could represent a therapeutic approach for ATLL patients. The combination delivery of miRNAs with chemotherapy drugs might provide a promising strategy to overcome chemo-resistance. Different studies have shown that co-delivery of miRNA and chemotherapeutic agents are effective to inhibit tumor growth by targeting genes, which are involved in tumor cell proliferation and/or survival [177,178,179]. In addition, in combination with antitumor drugs, miRNAs might have an important role by targeting genes involved in drug resistance, thus overcoming the chemo-resistance in ATLL patients.
